# Modeling Abnormal Priming in Alzheimer's Patients with a Free Association Network

**DOI:** 10.1371/journal.pone.0022651

**Published:** 2011-08-01

**Authors:** Javier Borge-Holthoefer, Yamir Moreno, Alex Arenas

**Affiliations:** 1 Instituto de Biocomputación y Física de Sistemas Complejos (BIFI), Universidad de Zaragoza, Zaragoza, Spain; 2 Departamento de Fsica Teórica, Universidad de Zaragoza, Zaragoza, Spain; 3 Complex Networks and Systems Lagrange Lab, Institute for Scientific Interchange, Torino, Italy; 4 Departament d'Enginyeria Informàtica i Matemàtiques, Universitat Rovira i Virgili, Tarragona, Spain; University of Maribor, Slovenia

## Abstract

Alzheimer's Disease irremediably alters the proficiency of word search and retrieval processes even at its early stages. Such disruption can sometimes be paradoxical in specific language tasks, for example semantic priming. Here we focus in the striking side-effect of hyperpriming in Alzheimer's Disease patients, which has been well-established in the literature for a long time. Previous studies have evidenced that modern network theory can become a powerful complementary tool to gain insight in cognitive phenomena. Here, we first show that network modeling is an appropriate approach to account for semantic priming in normal subjects. Then we turn to priming in degraded cognition: hyperpriming can be readily understood in the scope of a progressive degradation of the semantic network structure. We compare our simulation results with previous empirical observations in diseased patients finding a qualitative agreement. The network approach presented here can be used to accommodate current theories about impaired cognition, and towards a better understanding of lexical organization in healthy and diseased patients.

## Introduction

In the last several years, the understanding of semantic memory impairments in Alzheimer's Disease (AD henceforth) patients has been an important subject of investigation. Patients with this neurological disorder suffer severe memory deficits. Focusing on semantic memory, there is converging evidence about the general symptoms. Studies on spontaneous speech, verbal fluency, spelling and numerous other tasks all point to a progressive breakdown of knowledge about words and the objects they represent. However there is not such a consensus when it comes to explain unexpected or paradoxical performance. A situation of this kind is found when AD patients are confronted with a semantic priming (SP) task and results are compared with controls.

Semantic priming (SP) is one of the most common procedures for experimentally investigate the structure of semantic memory. It has captured the attention of cognitive scientists because it pervades language-related cognitive tasks like naming or lexical decision. The fundamental mechanisms explaining SP lie at word retrieval from memory. In this paradigm, pairs of words (a *prime* and a *target*) are sequentially presented. The semantic relation (or lack of it) between these two words determines whether a semantic priming effect appears. A faster response indicates a larger effect, whereas a slower one represents low or null effect. Such effects are also found in the context of AD, but their magnitude is, in general, diminished. However, for pairs of words belonging to the same category (category-coordinates, e.g. *lion – tiger*), a paradoxical hyperpriming, i.e. above-normal priming, occurs. “Above-normal” implies, in this context, priming effects stronger than those of healthy control subjects. The hyperpriming effect is well documented in AD patients under the semantic priming paradigm [1]–[5], and it also appears in other circumstances (for instance, in semantic dementia, [6]). But the nature of this effect is still unclear. Some researchers hold that AD patients suffer a loss of information in the semantic store, whereas others point to the difficulty to access and process semantic information, see [7]–[9].

We intend to explore the latter hypothesis capitalizing on modern network theory [10]. The recent burst and success of network modeling is not limited to the traditional niches of this framework. Nowadays, networks pervade manifold fields of science, ranging from biology –spreading of diseases [11]–[13] or robustness of gene regulatory networks [14], for example– to social science –emergence of cooperative behavior [15] or the diffusion of information in socio-technical systems [16]. On the other hand, graph theory has also proved useful in the somewhat less explored field of cognitive sciences. Phenomena related to semantic storage and mechanisms operating on it such as language growth and child language development [17]–[20], lexical availability [21], semantic similarity and category formation [22], [23] and verbal fluency [24], [25] can be analyzed from a network perspective. Modern network thinking represents a methodological update that still retains the intuitive character of the original framework rooted in the influential computer model put forward in [26], [27] and further elaborated by Collins [28].

## Results

In this work, we use a graph theoretical approach to explain abnormal priming in AD. To this end, we first formulate a network-based approach to study semantic priming. Secondly, we introduce a mechanism inspired by percolation processes to explain the reported hyperpriming in Alzheimer's patients. Our analysis complements purely neurophysiological studies and provides a framework that can also be used to study other neuropathologies with cognitive degenerations. The results here presented are also valuable to design new neuropsychological therapies in early stage patients of Alzheimer's.

### Free Association Norms and Priming

**Figure 1:**
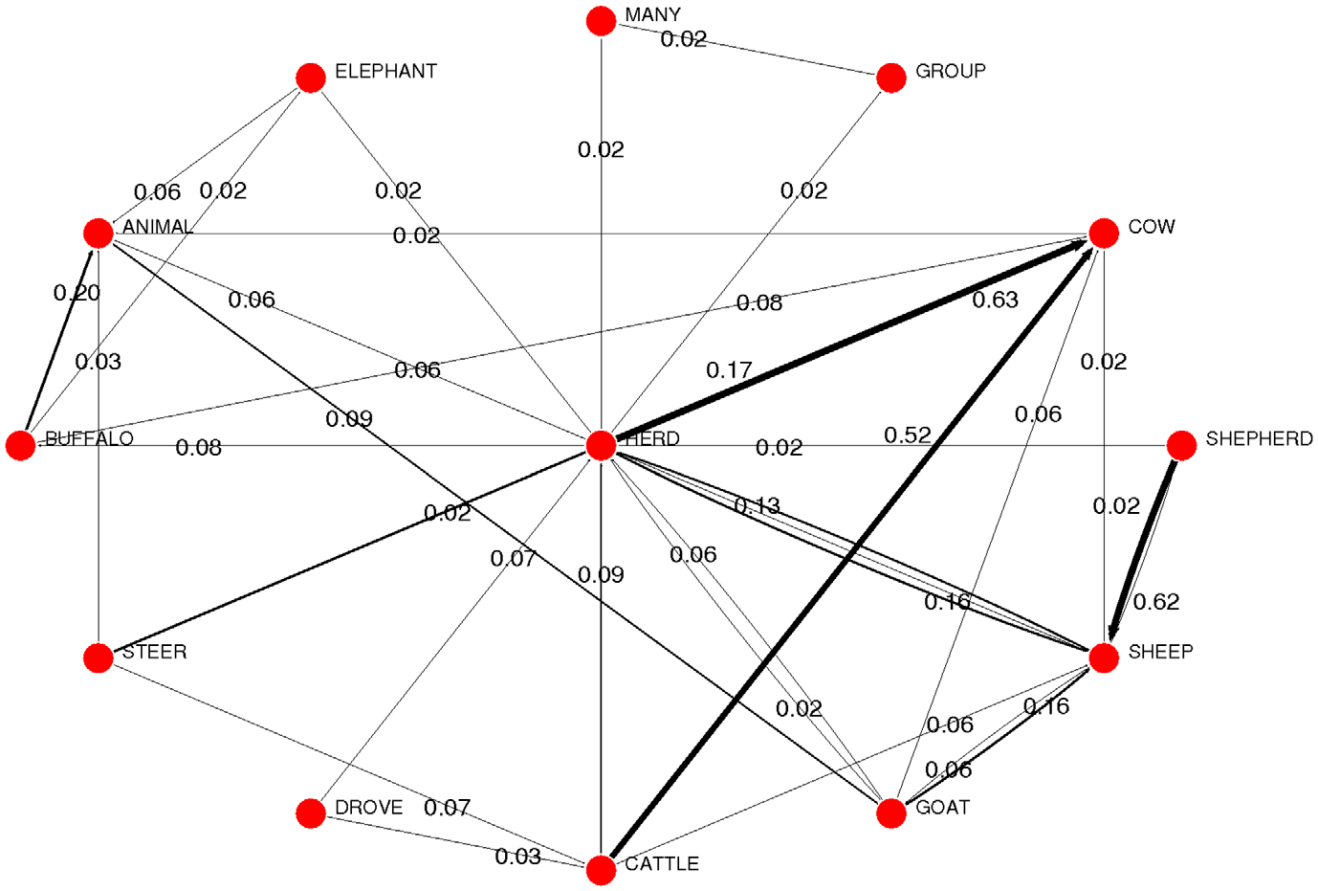
Partial representation of the Free Association network topology. Each node has a set of outgoing and incoming links. Because outgoing links correspond to produced frequencies, the resulting graph can be interpreted in terms of probabilities, i.e. the summation of HERD's outgoing links is 1.



**Figure 2:**
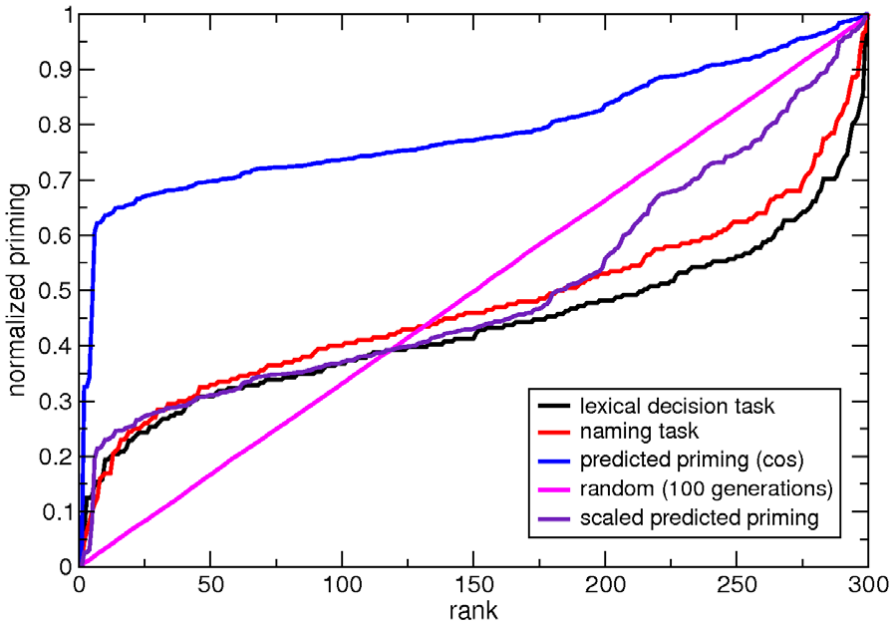
Modeling semantic priming. Comparison between priming as given by the closeness measure, Eq. (1), in association graphs and time in experiments involving naming or lexical decision tasks [35]. The curves depict the normalized priming measures as a function of the rank of different word pairs. Both synthetic and experimental priming behave in a similar way. The curve for the synthetic predicted priming almost collapse into the experimental ones when is rescaled using 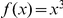.

Semantic networks are graphs in which vertices denote words, whereas links represent association relations. They can be constructed from many sources, be them text *corpora*, *thesauri* or psycho-linguistic data [23]. These structures share certain topological features which favor efficient search and retrieval processes –for example, “small-worldness” [29] or a power-law distribution of connections [30]. In the present study we use experimental data to construct such a network of semantic relations (see Materials and Methods). In particular, we construct a semantic network from the University of South Florida Free Association Norms [31], which includes 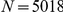 words or nodes, and take them as a proxy of the actual structure of semantic memory (see Materials and Methods). Free-Association networks are, by construction, directed and weighted. Weights represent the frequency of association in the sample, and their distribution is highly heterogeneous. The normalization of weights (frequencies) yields a probabilistic interpretation: the asymmetric adjacency matrix of the graph is a transition one, see Fig. 1. Semantic memory is the substrate for many cognitive dynamical processes. Here, we concentrate on semantic priming (SP). Although SP can be characterized in different ways –for example differences in event-related potentials–, in this work we refer only to reaction times. Thus in this work “priming effect” refers to empirical response-to-stimulus time scale. We hypothesize that priming effects, which are experimentally assessed in terms of *time*, can be explained in terms of *distance* in the association network. To show that our hypothesis actually holds, we measure such a distance in terms of *cosine similarity or closeness* [32], [33] between nodes. Topologically, cosine similarity between two words 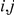 in the FA network expresses *structural* similarity, the proportion of common neighbors that these nodes have. Note that a direct link between such nodes does not add similarity, it rather decreases it given that no self-loops exist in the network. Let 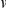 and 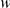 be the vectors reflecting the connectivity of nodes 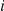 and 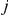 (i.e. the 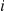
th and 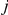
th rows in the network's adjacency matrix) respectively, the similarity 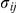 between nodes 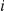 and 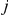 is

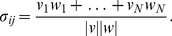
(1)There exists some evidence that association strength is at least partially responsible for priming effects [34]. However such evidence is controversial, and typically based merely on direct associations between prime and target, be them forward, backward or reciprocal relations. Here we show that our approach is actually compatible with empirical priming data, shifting attention from direct relations to common topological patterns between primes and targets. To this end we have calculated 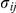 for every pair of words 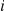, 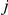 in the sample of semantic priming reported in [35]. For the sake of fidelity to the experiment, predicted priming is obtained as the difference between 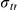 and 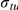, where 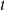 represents the target word, 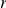 is the related prime, and 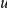 is the unrelated prime. Figure 2 represents empirical and synthetic priming results, normalized and sorted in increasing order. Experimental data includes two sets: priming results in a lexical decision task (the subject is asked to decide whether the presented stimulus is a word or a pseudo-word) and a naming task (subjects are asked to produce the stimulus aloud). It is apparent that these experimental distributions are very similar, which shows that the priming effect is consistent and robust across different tasks. On the other hand, we find a qualitative match, i.e., the same functional form, between experimental and predicted priming 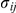. Admittedly, a rescaling of the curve corresponding to predicted priming in terms of distance almost collapses with those coming from experimental data (see Fig. 2). Thus, we conclude that the quantity 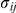 does grasp average aspects of real cognitive priming dynamics and can be used to translate time-related measures to distance-related properties on a topology.

### Semantic Network Degradation

**Figure 3:**
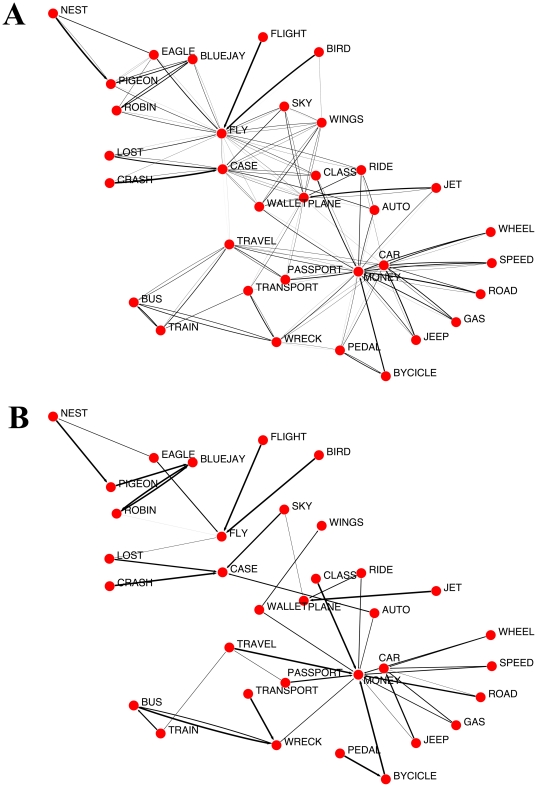
Structural global view of the degradation progress. Panel (a) correspond to the initial network (with no degradation), whereas panel (b) represents the damaged network when 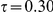. Two main consequences of the process are observed: the topology is impoverished as weaker links (thin lines) disappear, and at the same time some relationships are reinforced (thicker, darker lines) because of the probability normalization.

We next turn our attention to model the effects of Alzheimer's disease in cognitive processes by considering that the structural deterioration inherent to the disease affects the way words are interconnected in the semantic network as time goes on. Models of network damage are studied in the framework of percolation theory and typically consider random or targeted removal of nodes [36], [37]. The former strategy, often known as *error*, considers the failure (removal) of a node or link chosen at random; the latter, instead, *attacks* nodes (or links) which are considered important by virtue of some descriptor. For our purposes, neither of the two usual schemes are useful. We therefore introduce a novel form of structural deterioration specially suited to cognitive systems (referred to as degradation, henceforth). This new strategy is aimed to capture the physiological degrading processes in brain pathologies [38]–[42], which differ from attack (there is no selective action) and from error (which affects only one node/edge at a time). Degradation assumes that links are increasingly damaged. At a given threshold 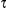, every link 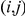 of the FA network is weakened, such that the new weight 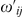 is given by 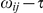. Note that the parameter 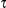 is seem as a measure of the disease progression in time: low values of 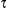 represent early stages of the disease, higher values correspond to later stages. Next, if 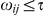, the link is removed. This process is performed for 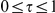 until degradation spans all possible weights in the topology. The out-links in the new, distorted structure are normalized, so as to maintain their probabilistic interpretation. Figure 3 illustrates the degradation process and the subsequent redistribution of associative strengths for 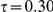. We limit the following analysis to values of 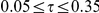, which correspond to mild-to-severe semantic memory damage, i.e., in a range that is still likely to be cognitively relevant (see Text S1). At the lower limit of this range we may assume that disease has begun its, though mildly, degrading action, whereas at the higher limit the remaining topology cannot hold cognitive activity anymore. We degrade the structure by increments of 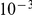 in 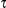 providing a high resolution of the whole process.

Link removal following degradation results in different predicted priming effects. This is evaluated, for each value 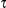, through the structural (cosine) similarity, Eq. (1), between every pair of words on the resulting, degraded structure. As previously shown, structurally close concepts would display a stronger priming effect than distant ones: higher closeness corresponds to a higher speeding of response time to target words. It is worth stressing that nodes that become disconnected due to the degradation process display no closeness to any node at all. Furthermore, the nodes that remain connected after a step in 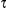 will increase their closeness due to the reinforcement of the surviving paths resulting from the normalization of the remaining links, which yields higher strengths. Under this probabilistic interpretation, abnormal increased closeness between words arises naturally.

### Synthetic versus Experimental Priming in Alzheimer's Disease

**Figure 4:**
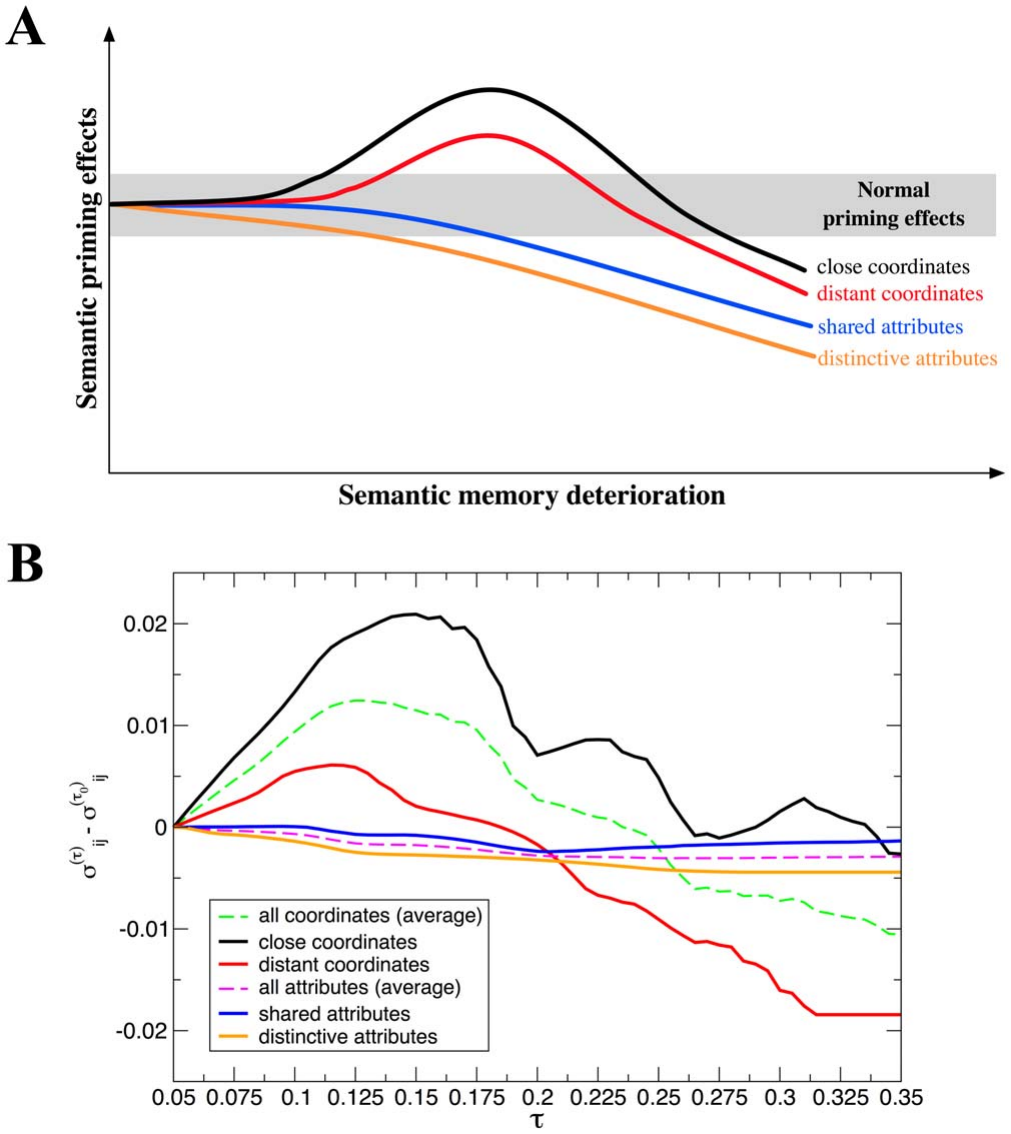
Semantic priming in damaged networks. **A**Schematic evolution of SP effects in relation to semantic memory degradation in AD (mild to moderate dementia). The figure is adapted from [5]. Lines illustrate the hypothetical evolution of the range of SP effects according to the semantic memory deterioration in different semantic relationship conditions: close and distant category-coordinate, shared and distinctive attribute. Average normal priming effect is represented by the grey area. Hyperpriming is illustrated by lines above it. An extinction of SP is observed in the AD patients in the shared and distinctive attribute conditions, with more severe vulnerability for distinctive attributes. Simultaneously, a hyperpriming effect was observed in both close and distant category-coordinate conditions, though to a lesser extent in the latter. **B**Average predicted priming (cosine similarity) results for the coordinate and attribute conditions for word pairs in [5]. The lists have been adapted such that the words belong to our empirical dataset –FA–. When this is not possible, other items have been chosen following the criteria in the original work. The list comprehends 72 pairs (18 under the label “close category-coordinates”, 18 in “distant category-coordinates”, 18 in “shared attributes” and finally 18 in “distinctive attributes”), see Text S1. The plot shows great resemblance to the upper panel for each condition: a transient hyperpriming effect appears before the whole performance decays.

Following the previous scheme, we evaluate the effects of a degradation process on structural similarity taking as a reference the list of words used in [5]. Figure 4a depicts in a schematic way the reaction-time results for priming in AD patients obtained in the aforementioned work, for different conditions. Category-coordinate stimuli are pairs of words which belong to the same semantic category. We can further refine this condition and distinguish close coordinates (*lion – tiger*, *cup – bowl*) from distant coordinates (*lion – whale*). The scheme compares semantic priming effects obtained in disease-free subjects and in patients at different stages of the disease (see Materials and Methods for more details). The hyperpriming phenomenon emerges in the early stage of the disease, and vanishes afterwards leading to the typical, well-known effects of Alzheimer's Disease on semantic memory, i.e. severe decay of performance. In the attribute condition the pair of words do not belong to the same category, they rather hold a part-whole relationship (*dog – tail*). Within the attribute condition, we can distinguish “shared attributes” (for instance, dogs, tigers and many other animals have a tail); from “distinct attributes”, those which are almost exclusive (for instance, zebras and stripes). Remarkably, different stimuli conditions (i.e. category-coordinates and attribute relations, and refinements therein) have a distinct response as the disease progresses.

The synthetic priming in Fig. 4b comes from degrading the Free Association network and monitoring the evolution with 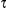 of the word pairs used for the lexical decision tasks (see Text S1), relative to initial synthetic priming, i.e. for 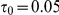. Results are depicted in Fig. 4b for both category-coordinate and attribute word pairs. The plot presents six traces: two of them are global averages for the coordinate (green) and attribute (magenta) conditions. The other curves represent further refinements of these categories as exposed in [5]. Also for those, the behavior of the coordinate conditions signals an early transient period of predicted hyperpriming –enhanced structural similarity– with a subsequent decay, finally falling below the initial level of SP. The hyperpriming effect is more remarkable in close coordinates. Despite the lack of significant statistics due to the limited length of the lists, they have been enriched by means of the production of synthetic samples (see Text S1). The results are in good qualitative agreement with the experimental semantic priming reported in [5], regarding both its functional shape and the relative magnitude of effects for every type of words pair under study. We provide further evidences of the validity of our approach in Text S1, where the same behavior is reported for other word pairs.

## Discussion

The method developed above tackles the study of semantic priming in Alzheimer Disease patients in two steps. We have first probed the capacity of a network approach to predict “standard” (healthy) semantic priming. Then, we have put forward the degradation scheme to link structural damage on the semantic network to disturbed performance, even when such performance is paradoxically enhanced. We now turn to analyze in detail the implications of the success of our proposal. Indeed, Fig. 4b evidences striking similarity to the ones reported from experimental works regarding hyper- and hypopriming. This qualitative agreement leads to two strong conclusions: (i) the hypothesis by which semantic deficits in AD stem from the difficulty to access and process semantic information is supported. The predictive success of our computational model is based on the idea that links are increasingly damaged, which is equivalent to hinder accessibility and proper navigation on the semantic network; and (ii) the so-called “category-coordinate condition” and “attribute condition” can be better understood in topological terms. Assertion (i) is supported by results: hindered accessibility, modeled as a degrading process of the connections of a network, stands as a sufficient condition to observe synthetic hyperpriming. This is compatible with a scenario in which semantic search and retrieval strategies are qualitatively the same, but occur in a distorted topology. We do not claim, however, that other malfunctions (e.g. cognitive slowing or concurrent word representation –node– damage [9]) might be also present in the emergence of abnormal priming effects. As for assertion (ii), close and distant coordinates can be defined in terms of *topological patterns*, overcoming merely intuitive definitions, or one based on formal oversimplifications.

**Figure 5:**
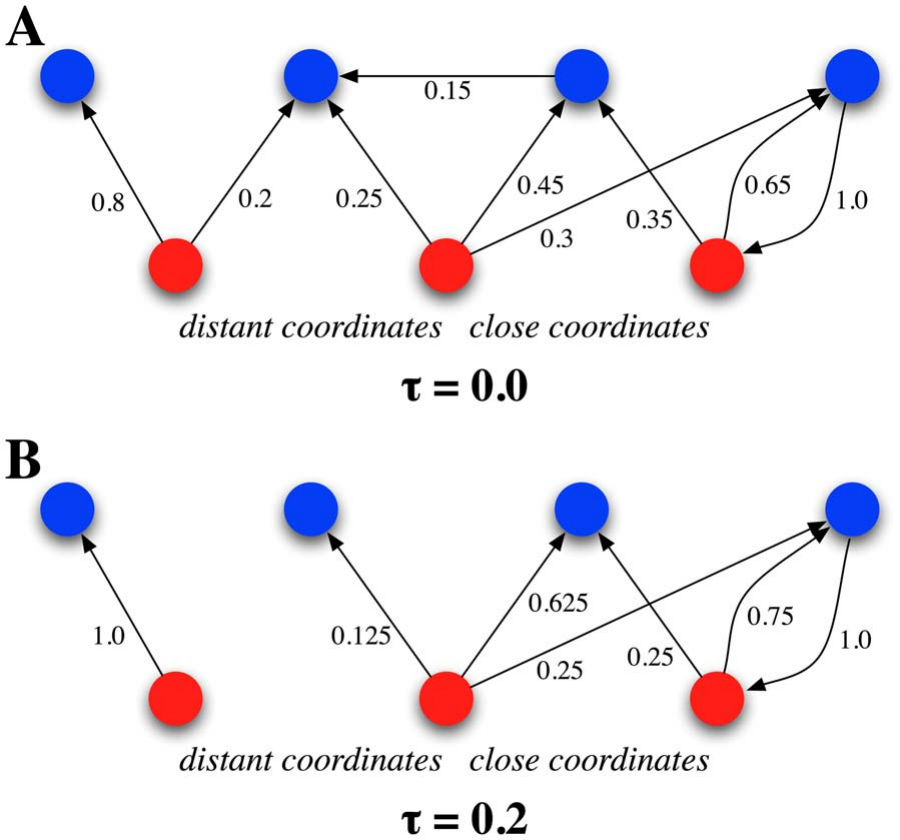
Coordinate condition scheme. Simplified scheme of degradation in the coordinate condition. From a topological perspective, close coordinate words (central and right red nodes) have a significant proportion of common neighborhood. Moreover, relations in such neighborhood are similarly strong, i.e. capable of surviving at least early degradation. On the other hand, distant coordinates (central and left red nodes) share a lesser amount of neighbors. Note that all weights have been weakened after applying a threshold 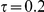; those links weaker than this threshold have been removed.

Following the sketch of SP effects as a function of semantic memory damage in [5] (Fig. 4a), our synthetic model offers an explanation for each case (close and distant coordinates, shared and distinctive attributes). Figure 5 illustrates a structural explanation for both close and distant coordinates. For these specific cases, hyperpriming is reported, being the effect more acute for close coordinates. The latter pairs typically share many associates in FA, their semantic proximity favors the fact that they are linked to some common attributes and to other coordinates in the semantic network. Topologically speaking, regardless of the fact that they have a direct, mutual connection, there usually exist many other paths connecting a coordinate pair of words, which implies a great deal of common neighbors. This entails that the degradation process does not affect such stimuli until deterioration is in a late stage. Since the remaining weights of links are normalized after the network has been thresholded, these tend to grow up to the moment when they disappear. This re-normalization implies a reinforcement in terms of the cosine similarity, thus the increase in synthetic priming is expectable up to mid-values in 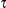 (Fig. 4). Beyond such values, common relationships are not held anymore, naturally accounting for the transient nature of the hyperpriming effect. On the other hand, although distant coordinates share many characteristics with close coordinates (see Fig. 5), the number of shared neighbors of the former is not as high as for the latter. Moreover, shared neighbors do not hold as strong relationships as in the coordinates case. This disparity in their connection patterns naturally yields a limited and smaller hyperpriming effect on such type of word pairs.

**Figure 6:**
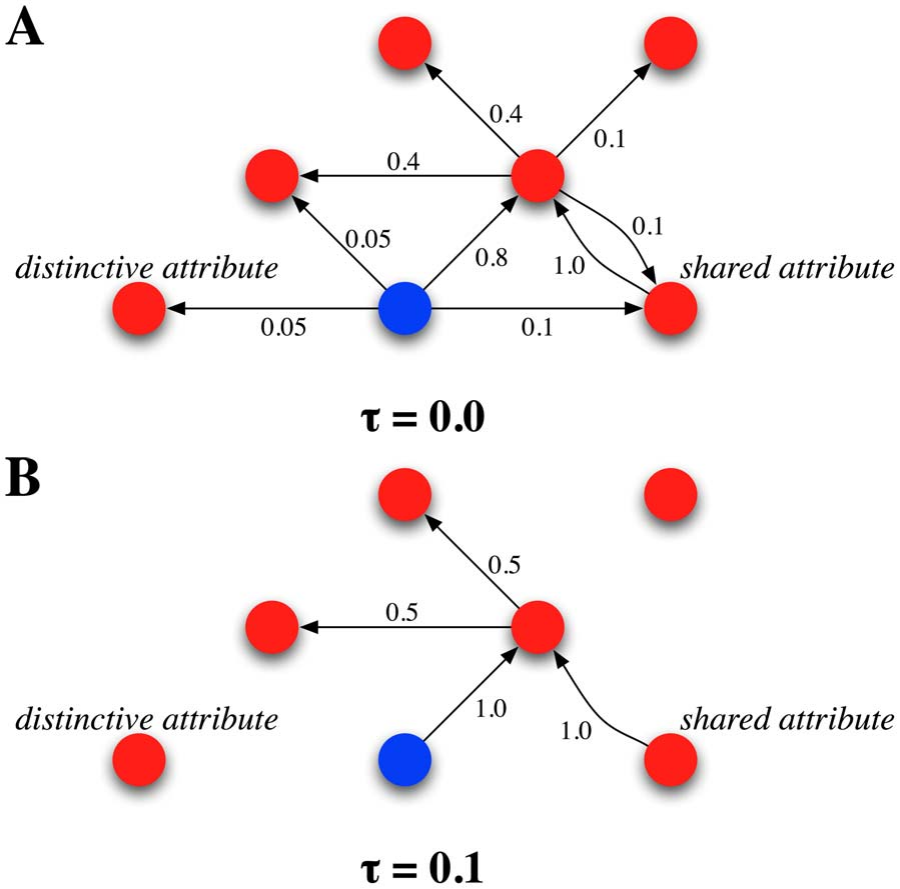
Attribute condition scheme. Topological characterization of the attribute condition, in an illustrative manner. Shared attributes exhibit a richer connectivity than distinctive attributes. This implies that complete disconnection typically appears at higher thresholding values. In the example, node 4 is a shared attribute for nodes 1 and 2. This fact explains the sustained evolution of closeness in the shared attribute condition, in contrast to the early collapse of the distinctive attribute condition (in the example, node 3).

The explanation for distinctive attributes is specially simple and elegant. By definition, these attributes are connected to very few concepts, because they are almost unique to those concepts. Being this so, degradation affects them enormously: as soon as the threshold achieves a certain value, the corresponding attribute's node becomes completely isolated, impeding the implicit spreading activation. This sharp dichotomy between existence/non-existence of a link is smoothed by statistics, in which distinctive attribute word pairs exhibit different weights, thus decay does not occur suddenly. Fig. 6 illustrates this phenomenon. The tendency for such word pairs is a slow decay in early stages of AD, and similarity dies out as early as 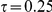. Furthermore, shared attributes show a similar pattern of decay compared to distinctive ones. Their decline, however, is not as fast. As it is apparent from Fig. 6, the main difference between distinctive and shared attributes is, in topological terms, the creation of triangles (clusters). Unlike distinctive attributes, then, the rupture of a direct connection between a concept and an attribute does not imply the complete disappearance of a SP effect, due to shared connections. Thus degradation affects distinctive attributes first and then shared ones.

The detailed explanation of each case from a structural perspective is compatible with the hypothesis that, not being exactly the same, hyperpriming is close to repetition priming [4], in which prime and target are the same. The loss of distinctive connections at early stages of semantic memory deterioration turns two distinct concepts into very close, almost exact ones regarding their connectivity profile. In terms of the structure of the semantic network, the connection pattern (neighborhood) of a certain node which has lost many of its connections is almost exactly the same as that of another node which has also lost its distinguishing connections. Under this topological perspective, being close-to-synonym is not necessarily being mutually connected; rather two words become synonyms because they share the exact same neighborhood. Beyond the abnormal early stage of disease in which repetition priming appears, it is presumed that impoverishment of conceptual knowledge will prevail, as defining attributes become inaccessible, and the associational strength between related concepts weakened. Topologically, such associative strength actually vanishes. Again, we emphasize that word-word relationships can be redefined in terms of connectivity patterns.

The previous structural arguments find their coinciding neurological counterpart in [39]–[42]. These works report the fragmentation of neural networks in AD and other patients suffering similar neuropathologies (which agree with the relative weight reinforcement due to link degradation and removal) and the disappearance of long range connections within such networks. The study of how changes at the physical layer are mirrored at the cognitive one is increasingly becoming a most relevant research issue.

On more general grounds, the explanation of hyperpriming as the result of a degradation process of the semantic network substrate could also be used to design specific complementary therapies at the early stage of the Alzheimer's disease from a neuropsychological perspective. Those would rely on the reinforcement of the semantic network by inducing the formation of structural links between distant coordinates and distinctive attributes, for example. We devise that this can be done by exposing patients to a sequential learning process linking these, in principle distant, concepts. The structural improvement of the semantic topology will increase the resilience to degradation. We however stress that most of the word pairs do not have SP effect, either at the system's healthy state (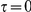) or in a distorted context. Additionally, hypopriming is the most common evolution. As degradation progresses, the main consequence must be a general impoverished performance, and hyperpriming is a rather restricted phenomenon, a collateral effect in a general semantically deficient scenario.

Finally, topological degradation is here assumed to be uniform, i.e., the threshold parameter acts upon *any* connection. This strategy suits computational modeling of general deterioration, as it is observed in diseases where global cognitive breakdown is observed. However this does not hold for much evidence, from which it is known that some parts of semantic knowledge might be deteriorated, whereas others remain undamaged [43]–[45]. Although selective damage has not been implemented in this work, it can be easily deduced that word pairs whose connections are not damaged do not yield unexpected phenomena, such as hyperpriming. This fact agrees with the ideas in [2] and [8], who report that hyperpriming is particularly noticeable for those items that explicit memory tasks had revealed to be degraded, but equivalent priming effects for patients and controls were found for items that were not degraded. Given the highly modular structure of Free Association [23], [46], some kind of selective degradation scheme could be designed such that different deterioration scenarios could be studied.

## Materials and Methods

### Free Association Norms

Experience with words creates a complex networked structure. Networked, because it is associative in nature: words are represented as vertices, association relations can be viewed as links. Complex, because edges are heterogeneous by construction: they may grasp any relation between words e.g. a causal-temporal relation (*fire* and *smoke*), an instrumental relation (*broom* and *floor*) or a conceptual relation (*bus* and *train*), among others. Because of the general character of association data, we take them as a proxy of the actual structure of semantic memory.

In practice, nodes and their links are obtained in cognitive-linguistic experiments. The best known Free Association data set in English are University of South Florida Free Association Norms (FA from now on; [31]). Nelson *et al.* produced these norms by asking over 6000 participants to write down the first word (*target*) that came to their mind when confronted with a *cue* (word presented to the subject). The experiment was performed using more than 5000 cues. Most of these words are nouns (76%), but other parts of speech are represented also: adjectives (13%) and verbs (7%). In addition, 16% are identified as homographs.

Among other information, a frequency of coincidence between subjects for each pair of words is obtained. As an example, words *car* and *road* are neighbors in this database, because a large fraction of the subjects related this target to this cue. Then, a directed and weighted network can be naturally constructed from the cue-target and frequency-counting schemes. The normalization of weights (frequencies) yields a probabilistic interpretation: the asymmetric adjacency matrix of the graph is a transition one, see Fig. 1. A network model of FA is the natural way to map the connections among words learned as a result of everyday experience, identifying the strength, number and direction of connections.

### Empirical hyperpriming

For the sake of clarity, we next briefly describe the experiment conducted with AD patients in [4]. We encourage the reader to look for more details in the original source. Empirical data regarding semantic priming performance in AD patients was obtained from 24 diseased subjects and 20 elderly normal controls. Both groups were 71 years old on average. AD patients were tested with a lexical decision task four times, every 6 months approximately. Thus, the whole evaluation spanned 18 months. Normal control subjects were tested once with the same protocol [4]. Priming effects were drawn from the lexical decision task, in which pairs of words were selected according to their semantic relation (coordinate relation, e.g. *tiger*- *lion*; attribute relation, e.g. *zebra*- *stripe*). This distinction was fine-grained further in [5]: coordinate relations were subdivided into ‘close’ and ‘distant’, whereas ‘shared’ and ‘distinctive’ attribute relations were distinguished. Other tasks (semantic knowledge, dementia severity index) were used to assess the deterioration effects of AD between sessions. In this work, we offer an explanation for paradoxical effects both in the coarse- and fine-grained versions of the experiment.

## Supporting Information

Text S1 **Provides supplementary insight to the main text's messages.** On one hand, it develops further the concept of network degradation and explores the structure's robustness. On the other, the main text closely follows experimental results that report hyperpriming; thus our theoretical framework has scarce empirical evidence to compare with. Text S1 synthetically enlarges the dataset on which hyperpriming can be found, by using another psycholinguistic data source. Finally, some statistical facts are highlighted so as to remark the that enhanced performance (hyperpriming) appears only as a transient phenomenon for a very specific experimental condition (category coordinates), in a general context of cognitive deterioration.
(PDF)
