# Supplementary material for: Modeling Abnormal Priming in Alzheimer's Patients with a Free Association Network
Source: PLoS One. 2011 Aug 1;6(8):e22651. doi: 10.1371/journal.pone.0022651 (PMC3148236; doi:10.1371/journal.pone.0022651)
Supplement: Text S1 — Provides supplementary insight to the main text's messages. On one hand, it develops further the concept of network degradation and explores the structure's robustness. On the other, the main text closely follows experimental results that report hyperpriming; thus our theoretical framework has scarce empirical evidence to compare with. Text S1 synthetically enlarges the dataset on which hyperpriming can be found, by using another psycholinguistic data source. Finally, some statistical facts are highlighted so as to remark the that enhanced performance (hyperpriming) appears only as a transient phenomenon for a very specific experimental condition (category coordinates), in a general context of cognitive deterioration. (PDF) [file pone.0022651.s001.pdf]

Supplementary Information
Degradation of a Free Association graph explains abnormal priming in Alzheimer's patients

Javier Borge-Holthoefer, Yamir Moreno, and Alex Arenas

April 17, 2011

Contents

1 Degradation process. 2

2 Results' validity, sensitivity and robustness. 2
2.1 Enlarging the test set: Feature Production Norms. 2
2.2 Enlarging the number of topologies: statistical errors. 3

3 Statistical significance of hyperpriming. 4

1 Degradation process.

Degradation assumes that links are increasingly damaged. At a given threshold τ, every link (i, j) of the underlying network is weakened, and the new weight is given by $\omega_{ij} - \tau$. All the weights corresponding to a given node i are then renormalized to keep their probabilistic meaning. The process is repeated adiabatically increasing τ at small intervals until degradation spans all possible weights in the topology. Fig. 1 illustrates the degradation process and the subsequent redistribution of associative strengths for two arbitrarily chosen networks.

As explained in the main text, the degradation parameter τ can be understood as a time scale: higher thresholding values correspond to later stages in semantic memory degradation. However, it is not possible to establish an exact mapping between τ and disease progression. Thus it is important to get a picture of how the topology behaves under degradation. To this end, we compute the *giant component* (GC) of the FA network. The GC of a network is the largest connected subnetwork. If a network is connected, the giant component's size N_{giant} equals the size of the system N_{net}. Figure 2 shows the evolution of the fraction of nodes, $\frac{N_{giant}}{N_{net}}$, that belongs to the GC of the FA network under degradation when τ is increased. The results indicate that the structure remains mostly undamaged up to $\tau = 0.1$. In the other extreme, FA's giant component is only a 5% of N_{net} at $\tau = 0.3$. For this reason, our degradation analysis considers only values from $\tau = 0.05$ to $\tau = 0.35$, which correspond to mild-to-severe semantic memory damage.

2 Results' validity, sensitivity and robustness.

In order to test our model against empirical data, the predicted priming for the list of word pairs in [1] has been monitored (see main text). The specific word pairs are listed in Table 1. Although the number of word pairs is quite small, statistics are reinforced with many subjects (both patients and controls) in the experimental setup. However, in our computational approach we only have at hand one topology (FA) on top of which the simulated degradation and dynamics are performed. Thus, we have devised two ways to overcome this limitation.

2.1 Enlarging the test set: Feature Production Norms.

The first option aims to reinforce our results enlarging the number of word pairs both in the category-coordinate and the attribute conditions. To do so, we make use of public psycholinguistic data. Feature Production Norms (FP) were collected by [2] by asking subjects to conceptually recognize features when confronted with a certain word. This feature collection is used to build up a vector of characteristics for each word, where each dimension represents a feature. In particular, participants are presented with a set of concept names and are

asked to produce features they think are important for each concept. Each feature stands as a vector component, with a value that represents its production frequency across participants. These norms include 541 living and nonliving concepts, for which semantic similarity is computed as the overlap between pairs of vectors of characteristics. The rationale behind this collection is quite intuitive: the more features two concepts share, the more semantically similar they must be.

Accordingly, a significant amount of category-coordinate word pairs can be obtained using FP, by filtering those pairs of words which display significant similarity. In this fashion we have obtained 247 extra pairs of category-coordinate words, which correspond to words in FP exhibiting a similarity greater than 0.65. On the other hand, we have filtered features in FP to retain the ones under the label "visual-form and surface" and such that the feature's rank is below 3, i.e. the feature has at most rank 3 within the concept according to production frequency. In this way a list with 244 extra object–attribute pairs has been obtained. Needless to say, we have constrained the data mining such that words in these new lists are also present in FA.

Fig. 3 depicts the average evolution of semantic priming taking as a reference the lists obtained from FP. As in the case of empirically tested lists (see main text), results evidence a qualitative resemblance to those reported in [3]. Regarding the coordinate condition hyperpriming emerges in the early stage of degradation, and vanishes afterwards (black solid line). The attribute-condition list evidences similarity to empirical results for low thresholding values, but clearly deviates at the end (red solid line). Nonetheless, it must be taken into account that the longitudinal study in [3] included only four experimental sessions. Thus the general tendency in both coordinate and attribute conditions can be recovered, see dashed lines in Fig. 3.

2.2 Enlarging the number of topologies: statistical errors.

The FA norms are surely exposed to several sources of experimental errors which are unobservable. It is mandatory then to account for errors in the sample, which are understood as deviations of the sample from the (unobservable) true value. We analyze this using confidence intervals for the whole set of similarity values sampled. Standardizing the similarity values we determine that with a 95% confidence, the error is around 10% in the measure. Taking into account this value, we can generate synthetic samples of FA networks by using the original data and adding gaussian noise to the links with a variance of 10%.

The consideration of statistical errors allows us to overcome the strong constraint on the number of "synthetic subjects" on which priming may be predicted: different FA realizations account for individual differences in the structure of semantic network. This methodology has proved to smooth the behavior of the functions represented without changing its qualitative structure. Furthermore, the introduction of variability in the underlying topology yields valuable hints on the sensitivity of our proposed measures and guarantees the robustness of the results. Both Fig. 4b (main text) and Fig. 3 have been ob-

tained averaging predicted priming over 21 topologies, that is, the original FA plus 20 alternative topologies following the procedure described in the previous paragraph.

3 Statistical significance of hyperpriming.

Beyond any doubt, hyperpriming in AD patients is a side-effect of the ongoing disease. Enhanced performance appears as a transient phenomenon for a very specific experimental condition (category coordinates), in a general context of cognitive deterioration. Being this so, our computational account must also reflect these facts.

Following the structural approach developed so far, we can try to detect pairs of words which present an abnormal, transient growth in their predicted priming. Also, once the degradation process arises as a plausible explanation for hyperpriming, it is possible to use our model as a predictive tool. In this line, we have monitored across τ the whole set of possible pairs in our empirical data, to check for atypical closeness (our SP's proxy) evolutions. Out of the possible $N \times (N-1)$ pairs, we only monitor those pairs in which predicted priming when degradation begins $\sigma_{ij}^{\tau_0} > 0$. Seeking for early increases, we have detected 73,012 pairs (3.1% of the total possible) which display hyperpriming; 2,218,070 (96.9%) pairs match the hypopriming scheme. These figures match our expectations: (i) most of the word pairs have no SP effect, either at the system's healthy state (τ_0) or in a degraded context; (ii) hypopriming is the most common evolution. As degradation progresses, the main consequence must be a general impoverished performance; and (iii) hyperpriming is a rather restricted phenomenon, a collateral effect in a general semantically deficient scenario. Furthermore, a close look to the list of word pairs with predicted hyperpriming expands the range of words which might display this phenomenon beyond category-coordinates (see Table 2 for some examples). This would indicate that there exists a class of words which share a similar connectivity profile. Within this class one may find category-coordinates, but just as well other types of relations. This novel, more general insight reveals, as expected, that the complexity in the organization of semantic knowledge is far beyond the *ansatz* in [4].

References

[1] Laisney M, Giffard B, Belliard S, de la Sayette V, Desgranges B, et al. (2009) When the zebra loses its stripes: Semantic priming in early Alzheimer's disease and semantic dementia. Cortex doi 10.1016/j.cortex.2009.11.001.

[2] McRae K, Cree G, Seidenberg M, McNorgan C (2005) Semantic feature production norms for a large set of living and nonliving things. Behav Res Methods 37: 547-559.

[3] Giffard B, Desgranges B, Nore-Mary F, Lalevee C, Beaunieux H, et al. (2002) The dynamic time course of semantic memory impairment in Alzheimer's disease: clues from hyperpriming and hypopriming effects. Brain 125: 2044.

[4] Collins AM, Quillian MR (1969) Retrieval time from semantic memory. J Verbal Learn Verbal Beh 8: 240-247.

Table 1: Monitored word pairs from which results in Fig. 4 of the main text have been obtained. In italics, word pairs that have been adapted to acommodate them to available empirical data (FA).

Distinctive attribute	Shared attribute	Distant category-coordinate	Close category-coordinate
train-wagon	pigeon-wings	spoon-fork	wardrobe-table
bicycle-pedal	gorilla-hair	*comb-brush*	whale-shark
spider-web	boot-heel	bee-wasp	*ant-beetle*
pine-needle	*bicycle-wheel*	*tiger-lion*	plate-bowl
shoe-lace	duck-feather	*jeans-trousers*	lobster-shrimp
stag-woods	cap-fabric	strawberry-raspberry	chair-bench
glasses-frame	cat-whiskers	*pear-apple*	*lettuce-celery*
plane-wings	*crow-wings*	pony-horse	fly-butterfly
dog-bark	pick-handle	sandals-shoe	truck-tractor
zebra-stripe	scissors-blade	*bison-buffalo*	daisy-tulip
hammer-head	tiger-claw	cup-bowl	*nail-screw*
bed-sheet	brush-bristle	garlic-onion	cow-sheep
crab-claw	lizard-tail	desk-table	knife-saw
sheep-wool	palm-trunk	snail-slug	dandelion-daisy
mill-blade	cherry-stem	wolf-dog	trousers-shirt
elephant-trunk	grasshopper-leg	necklace-bracelet	*peach-strawberry*
basket-wicker	pumpkin-seed	corn-wheat	vase-glass
snail-shell	*oak-leaf*	*tie-scarf*	*stag-pig*

Table 2: Some of the pairs of words with predicted hyperpriming. Note that a notable proportion of pairs are category-coordinates (for instance, all the examples with planets involved), but the phenomenon appears also in a more general context (such as in *pasta-mafia* or the pairs in which *fragile* appears).

shadow-midnight	frequency-television	pasta-sauce	Venus-Saturn	fragile-fix
shadow-alley	frequency-speaker	pasta-noodles	Venus-Jupiter	fragile-bend
shadow-light	frequency-frequent	pasta-mafia	Venus-Pluto	fragile-smash
shadow-tan	frequency-seldom	pasta-meatballs	Venus-Uranus	fragile-fracture

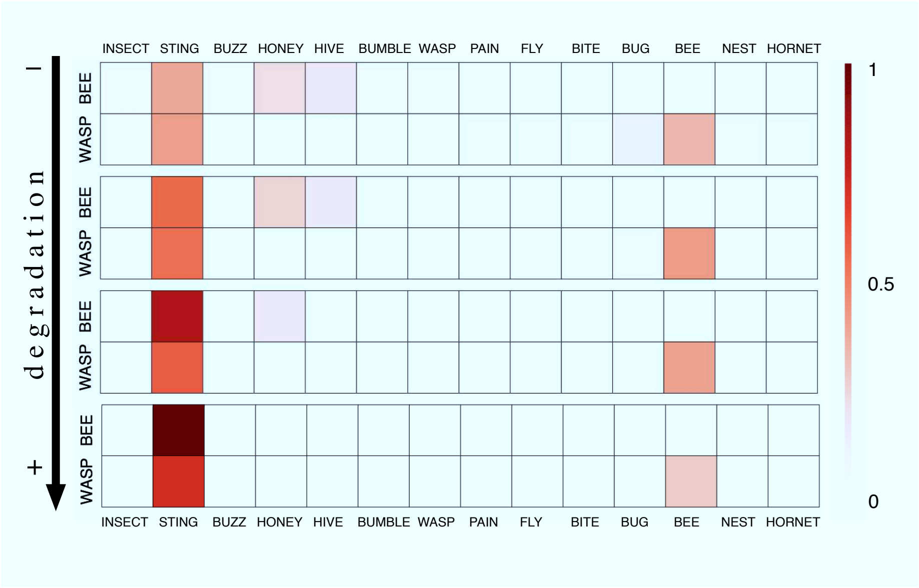

Figure 1: Structural local view of the degradation progress: The neighborhoods for the words BEE and WASP are represented as degradation takes place. The value of the threshold τ increases from top to bottom (0.0, 0.1, 0.2 and 0.3 respectively), the weaker connections are progressively removed. Due to normalization, the surviving links increase their value. Note that the word BEE progressively looses its connections towards HONEY or HIVE; the connection from WASP to BUG is also lost. The consequence is a reinforcement of BEE and WASP respective connections to STING, which in the end will promote a higher closeness between both words. The paradoxical growth in the words' mutual overlap (cosine) is naturally explained through the described topological change, see text for details.

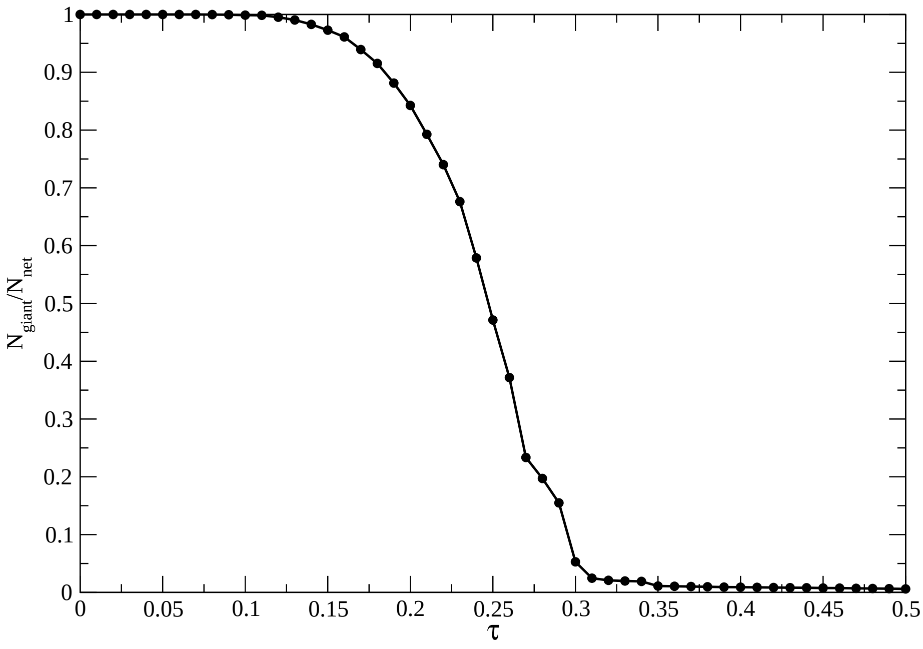

Figure 2: Average evolution of the SP effect, both in the coordinate (small black circles) and the attribute (small red squares) condition. The plot begins at $\tau = 0.05$ to emulate AD's early stages (an already damaged structure); and ends at $\tau = 0.35$, when the FA topology is already disintegrated. Discontinuous thick lines (black and red) show the tendency in SP if four single arbitrary points (t1, t2, t3 and t4, green vertical lines) are taken into account, recalling real experimentation in [3] which included only four experimental sessions.

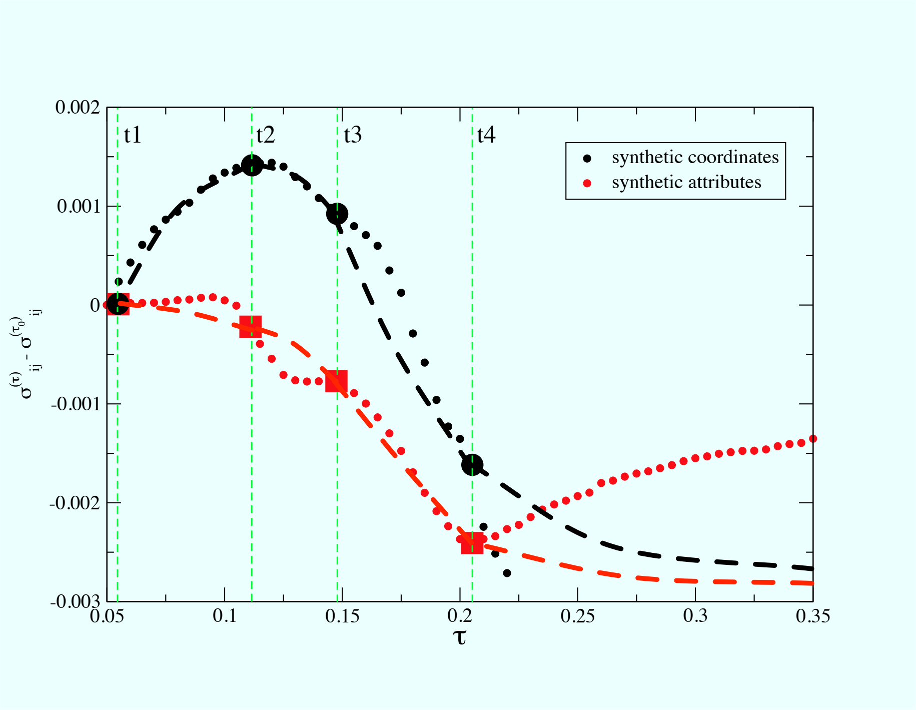

Figure 3: Average evolution of the SP effect, both in the coordinate (small black circles) and the attribute (small red squares) condition. The plot begins at $\tau = 0.05$ to emulate AD's early stages (an already damaged structure); and ends at $\tau = 0.35$, when FA topology is already disintegrated. Dashed thick lines (black and red) show the tendency in SP if four single arbitrary points (t1, t2, t3 and t4, green vertical lines) are taken into account, recalling real experimentation in [3] which included only four experimental sessions.
